# Exploring the influence of track and field athletes' self-management on athletic performance: the chain mediating role of psychological resilience and competitive spirit

**DOI:** 10.3389/fpsyg.2026.1934680

**Published:** 2026-10-22

**Authors:** Zhengzheng Fan, Yuanzhen Ni, Yibo Shen

**Affiliations:** 1 College of Physical Education and Sport Science, Qufu Normal University, Qufu, China; 2 School of Physical Education, Liaocheng University, Liaocheng, China; 3 Sports and Artificial Intelligence, School of Physical Education, Shandong University, Jinan, China; 4 Department of Physical Education, Kyungpook National University, Daegu, Republic of Korea

**Keywords:** athletic performance, chain mediation, competitive spirit, psychological resilience, self-management, track and field athletes

## Abstract

**Objective:**

Against the backdrop of increasingly intense competition in competitive sports, the mechanisms underlying the improvement of athletes' athletic performance have become an important research topic. Based on self-regulation theory and relevant perspectives in sport psychology, this study explores the relationship between self-management and athletic performance among track and field athletes, and examines the single mediating and chain mediating effects of psychological resilience and competitive spirit.

**Methods:**

This study employed a cross-sectional quantitative survey design and used non-probability convenience sampling to recruit 772 Chinese track and field athletes. Established scales were used to assess self-management, psychological resilience, competitive spirit, and athletic performance. SPSS 31.0 and AMOS 31.0 were used for descriptive statistics, correlation analysis, common method bias testing, confirmatory factor analysis, and structural equation modeling. Direct, individual indirect, and serial indirect effects were examined using 5,000 bootstrap samples.

**Results:**

Self-management was positively associated with athletic performance. Psychological resilience and competitive spirit each significantly mediated the relationship between self-management and athletic performance. In addition, psychological resilience and competitive spirit formed a significant serial mediating pathway, indicating that higher self-management was associated with greater psychological resilience, which was subsequently associated with stronger competitive spirit and, ultimately, better athletic performance.

**Conclusion:**

Self-management is an important factor influencing the athletic performance of Chinese track and field athletes. Its effect is reflected not only in its direct promotion of athletic performance but also in its indirect effect through the sequential psychological mechanism of psychological resilience and competitive spirit. The findings suggest that improving the athletic performance of track and field athletes should not focus solely on sport-specific technical and physical training, but should also emphasize the cultivation of self-management ability, psychological resilience training, and the shaping of psychological goals related to competitive spirit.

## 1 Background

Athletic performance is a central outcome in competitive sport and reflects athletes' ability to translate physical preparation, technical skills, and psychological readiness into effective competitive execution. In track and field, prolonged training cycles, high physical demands, competitive pressure, and fluctuations in performance make psychological and behavioral regulation particularly important. Recent research has increasingly emphasized the contribution of psychological and self-regulatory factors to athletic performance alongside traditional physical and technical determinants (Reinebo et al., 2024).

Among these factors, self-management enables athletes to regulate training behavior, physical condition, psychological state, and daily routines, thereby supporting consistent preparation and performance (Kim and Cruz, 2021; Young et al., 2023). Psychological resilience helps athletes adapt to pressure, setbacks, and performance fluctuations and maintain effective functioning under adversity (Gupta and McCarthy, 2022). Competitive spirit, reflecting athletes' motivation to pursue achievement and competitive success, may further support sustained effort and performance-oriented behavior (Bugten et al., 2021). Thus, these variables represent complementary processes of behavioral self-regulation, psychological adaptation, and competitive motivation.

Although previous studies have demonstrated associations between self-management and performance-related outcomes, these variables have largely been examined separately. Consequently, less is known about whether self-management is associated with athletic performance through a sequential psychological process involving resilience and competitive spirit. From a self-regulation perspective, effective self-management may strengthen athletes' capacity to adapt to competitive demands, which may subsequently reinforce competitive motivation and be associated with better athletic performance.

Accordingly, this study examines the relationship between self-management and athletic performance among Chinese track and field athletes, with particular attention to the individual and serial mediating roles of psychological resilience and competitive spirit. The contribution of this study lies not simply in re-examining the self-management–performance relationship, but in integrating behavioral self-regulation, psychological adaptation, and competitive motivation within a single sequential framework.

### 1.1 The direct effect of self-management on athletic performance

From the perspective of self-regulation theory, athletic performance can be understood as a goal-oriented outcome, and its formation may be closely related to athletes' ability to plan, monitor, and adjust during training and competition (Zimmerman, 2000). Self-management reflects athletes' active regulation of training behaviors, physical condition, psychological state, and life rhythm. For track and field athletes, the training cycle is relatively long, training load is high, and athletic results are easily affected by fluctuations in physical and psychological states. Therefore, good self-management may help them maintain regular training, increase training commitment, and sustain a relatively stable competitive state.

Athletes with higher levels of self-management are generally more likely to show stronger initiative in training arrangement, physical recovery, emotional regulation, and state adjustment, and they are able to make timely adjustments to their own condition based on training feedback. Previous studies have shown that high-level athletes often possess strong self-regulation abilities and can more proactively plan, monitor, and reflect on the training process (Toering et al., 2009). Kim (2003) also pointed out that elite athletes tend to demonstrate relatively strict psychological management, training management, and physical management, indicating that self-management may be an important psychological foundation for maintaining a good competitive state. Therefore, athletes with higher levels of self-management may be more likely to maintain stable commitment, focused execution, and state regulation during training and competition, and further demonstrate higher levels of athletic performance.

- **H1:** Self-management has a significant positive predictive effect on athletic performance (**path a**).

### 1.2 The mediating role of psychological resilience

From the perspective of self-regulation theory, self-management may further influence athletes' athletic performance by enhancing their psychological resilience (Zimmerman, 2000). Psychological resilience is an important psychological resource that enables athletes to maintain positive adaptation, emotional stability, and goal persistence when facing stress, failure, injury, and competition (Fletcher and Sarkar, 2012). Athletes with higher levels of self-management are usually better able to plan training, monitor their physical and psychological states, and adjust coping strategies in a timely manner (Kim, 2003). Continuous self-management can strengthen athletes' sense of control over training and competition processes, making it easier for them to maintain a stable mindset and recovery ability in situations involving setbacks and stress. Therefore, the higher the level of self-management, the more likely athletes are to demonstrate stronger psychological resilience.

The improvement of psychological resilience further contributes to athletic performance. Track and field athletes often face high training loads, performance fluctuations, and competitive pressure during training and competition. Athletes with higher psychological resilience are better able to withstand pressure, regulate negative emotions, and maintain focus and stable performance in critical situations. Previous studies have shown that psychological resilience is an important psychological resource for athletes to achieve good athletic performance in high-pressure environments (Fletcher and Sarkar, 2012), and is closely related to athletic achievement and mental health (Hosseini and Besharat, 2010). Therefore, self-management may further improve athletes' athletic performance by enhancing psychological resilience.

- **H2:** Psychological resilience plays a single mediating role between self-management and athletic performance (**path bf**).

### 1.3 The mediating role of competitive spirit

Self-management may also be associated with athletes' athletic performance through competitive spirit. Athletes with higher levels of self-management are usually more likely to have clear training goals and competition goals, and they can maintain relatively stable competitive motivation through training management, psychological management, and physical management (Kim, 2003). From the perspective of self-regulation theory, individuals' initiative in goal setting, process monitoring, and behavioral adjustment may help strengthen their expectations for performance improvement and competition success (Zimmerman, 2000). Therefore, athletes with higher levels of self-management may be more likely to develop stronger competitive awareness and a stronger pursuit of victory.

Competitive spirit refers to athletes' psychological tendency to pursue victory, defeat opponents, and achieve excellent results in competitive contexts, reflecting strong competitive motivation, goal orientation, and outcome awareness (Gill and Deeter, 1988). For track and field athletes, the long-term repetitive training process and highly competitive competition environment require sustained goal persistence and competition commitment. A higher level of competitive spirit may help athletes maintain focus, withstand pressure, and actively strive for advantages during training and competition, thereby being associated with better athletic performance. Based on this, self-management may further influence track and field athletes' athletic performance by enhancing competitive spirit.

- **H3:** Competitive spirit plays a single mediating role between self-management and athletic performance (**path ed**).

### 1.4 The chain mediating role of psychological resilience and competitive spirit

From the perspective of a continuous psychological process, the influence of self-management on athletic performance may not be achieved through a single psychological variable, but through the sequential effects of psychological resilience and competitive spirit. Athletes with higher levels of self-management are generally better at planning training, monitoring their condition, and regulating stress. This helps improve their psychological resilience, enabling them to maintain stable adaptive capacity when facing training loads, competition pressure, and performance fluctuations (Kim, 2003; Zimmerman, 2000). Athletes with higher psychological resilience are more likely to perceive pressure and competition as challenges rather than threats, thereby further stimulating their competitive spirit and strengthening their motivation to pursue victory, persist in goals, and engage in active competition (Fletcher and Sarkar, 2012; Sarkar and Fletcher, 2014).

The sequential effects of psychological resilience and competitive spirit further contribute to athletic performance. Psychological resilience helps athletes maintain emotional stability and focus under stressful conditions, while competitive spirit transforms this psychological adaptability into stronger training commitment and competition execution. Previous studies have indicated that psychological resilience is closely related to protective factors such as motivation, confidence, and concentration (Sarkar and Fletcher, 2014), while competitive spirit reflects athletes' desire to pursue victory in competition (Gill and Deeter, 1988). Therefore, self-management may first enhance psychological resilience, then further improve competitive spirit, and ultimately promote athletes' athletic performance.

- **H4:** Psychological resilience and competitive spirit play a chain mediating role between self-management and athletic performance (**path bcd**).

### 1.5 Research model and path coding

Based on the above theoretical analysis and literature review, this study constructs the research model shown in Figure 1, aiming to explore the mechanism through which self-management influences track and field athletes' athletic performance and to examine the single and chain mediating roles of psychological resilience and competitive spirit.

**Figure 1:**
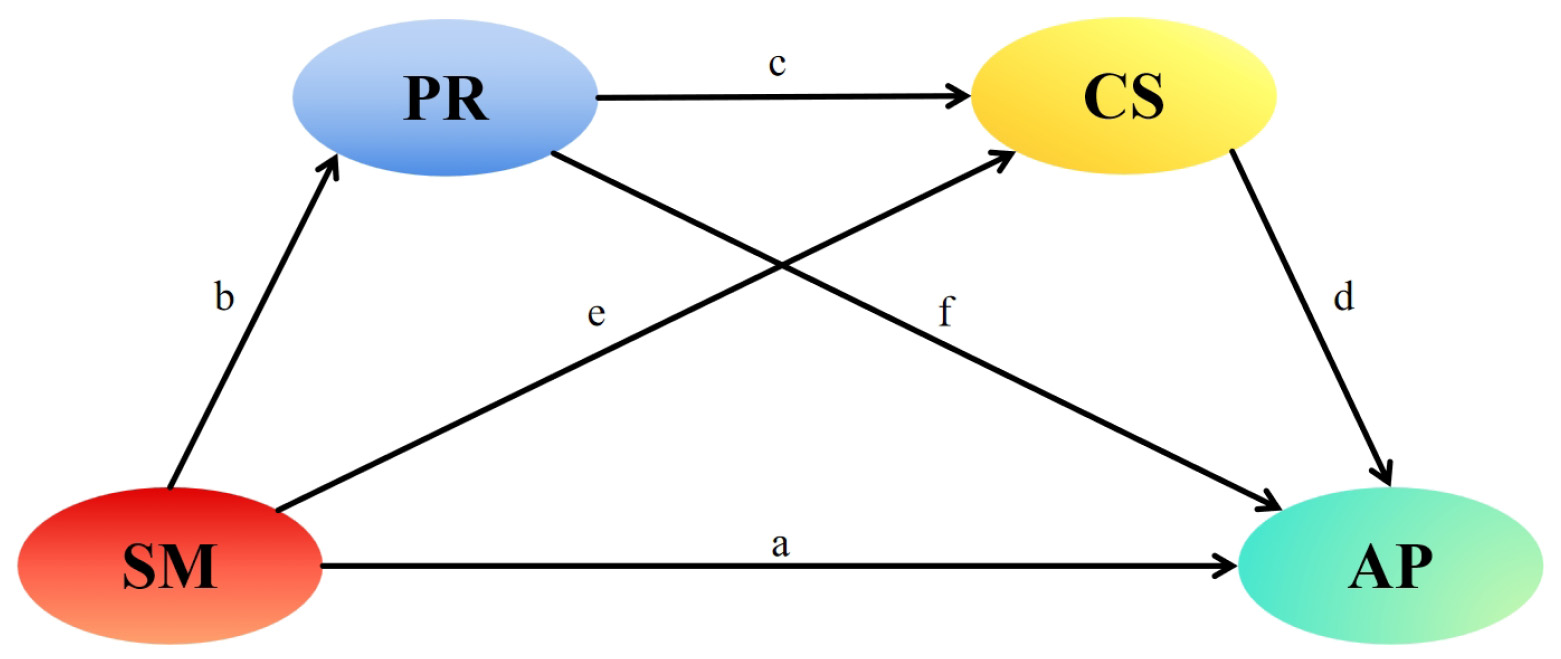
Research model. SM, Self-Management; PR, Psychological Resilience; CS, Competitive Spirit; AP, Athletic Performance. Path a represents the direct path from SM to AP; path b represents SM → PR; path c represents PR → CS; path d represents CS → AP; path e represents SM → CS; path f represents PR → AP. Based on these path labels, the indirect effect through PR was calculated as b × f, the indirect effect through CS as e × d, the serial mediation effect as b × c × d.

## 2 Methods

### 2.1 Participants

A non-probability convenience sampling technique was used to recruit Chinese track and field athletes (Golzar et al., 2022). Recruitment was conducted across Shandong, Liaoning, Jiangsu, Henan, Hebei, Hunan, and Hubei Provinces, as well as through track and field clubs in other regions of China. Participants were recruited from high schools, sports colleges, track and field teams, and clubs, with teachers and coaches assisting in distributing the questionnaire through Wenjuanxing (Questionnaire Star), a widely used online survey platform in China. To be eligible for participation, individuals were required to be currently engaged in track and field training and to participate voluntarily in the study.

The first page of the questionnaire explained the purpose and procedures of the study, the principles of anonymity and confidentiality, and participants' right to withdraw at any time. Participants proceeded to the formal questionnaire only after providing informed consent.

After data collection, responses were screened for questionnaire completeness, response time, patterned responding, and duplicate submissions. A total of 847 questionnaires were collected. After excluding 75 invalid responses, 772 valid questionnaires were retained for the final analysis, yielding an effective response rate of 91.1%.

Among the valid samples, 679 participants were male (88.0%) and 93 were female (12.0%). In terms of educational level, 597 participants had a high school education (77.3%) and 175 had a university education (22.7%). Regarding athletic level, 94 participants were first-level athletes (12.2%), 240 were second-level athletes (31.1%), 180 were third-level athletes (23.3%), and 258 were non-ranked athletes (33.4%). In terms of sport event, 685 participants were sprinters (88.7%) and 87 were long-distance runners (11.3%). Regarding years of training, 283 participants had trained for 1–2 years (36.7%), 221 for 2–3 years (28.6%), 117 for 3–4 years (15.2%), 47 for 4–5 years (6.1%), 34 for 5–6 years (4.4%), and 70 for more than 6 years (9.0%). The specific demographic characteristics are shown in Table 1. Given the composition of the sample, the findings should be interpreted primarily in the context of a predominantly male and sprint-oriented group of Chinese track and field athletes. In this study, “non-ranked athletes” referred to participants who did not hold an official athletic-level classification at the time of the survey.

**Table 1:** Demographic characteristics of participants.

Variable	Category	*n* (%)	Variable	Category	*n* (%)
Gender	Male	679(88.0%)	Event	Sprint	685(88.7%)
	Female	93(12.0%)		Long-distance running	87(11.3%)
Education level	High school	597(77.3%)	Training years	1–2 years	283(36.7%)
	University	175(22.7%)		2–3 years	221(28.6%)
Athletic level	Level 1	94(12.2%)		3–4 years	117(15.2%)
	Level 2	240(31.1%)		4–5 years	47(6.1%)
	Level 3	180(23.3%)		5–6 years	34(4.4%)
	No level	258(33.4%)		More than 6 years	70(9.0%)

### 2.2 Measurement instruments

This study used four established scales to assess self-management, psychological resilience, competitive spirit, and athletic performance. Based on the theoretical foundations and dimensional structures of the original instruments, each scale was translated, back-translated, and contextually adapted for Chinese track and field athletes. To establish content validity and contextual appropriateness, three experts in sport psychology, physical education, and sport measurement and evaluation independently reviewed the items in terms of content representativeness, linguistic accuracy, clarity of expression, and contextual relevance. Item wording was revised according to the experts' feedback before the final questionnaire was administered. The content validation process was qualitative; therefore, no quantitative content validity index was calculated. Subsequently, the reliability and construct validity of the final instruments were evaluated using Cronbach's α, composite reliability (CR), average variance extracted (AVE), and confirmatory factor analysis.

All scales adopted a 5-point Likert scoring method, ranging from 1 = “strongly disagree” to 5 = “strongly agree,” with higher scores indicating higher levels of the corresponding variable. Meanwhile, this study used confirmatory factor analysis to examine the structural validity of the measurement model, and evaluated the internal consistency reliability and convergent validity of each scale using Cronbach's α, composite reliability, and average variance extracted. The details are as follows.

#### 2.2.1 Self-management

The Athletes Self-Management Questionnaire (ASMQ) developed by Huh (2003) was used. The scale includes four dimensions: physical management, interpersonal relationship management, training management, and mental management, with a total of 18 items. Cronbach's α = 0.895.

#### 2.2.2 Psychological resilience

The Resilience Quotient Test (RQT) developed by Shin W. Y. et al. (2009) was used. The scale includes three dimensions: control, positivity, and sociality, with a total of 27 items. Cronbach's α = 0.931.

#### 2.2.3 Competitive spirit

The Competitive Spirit Scale (CSS) developed by Shin J. T. et al. (2009) was used. The scale includes three dimensions: fighting spirit, self-confidence, and desire to win, with a total of 18 items. Cronbach's α = 0.897.

#### 2.2.4 Athletic performance

The Athletic Performance Scale (APS) developed by Choi (2014), was used. The scale includes three dimensions: competition operation ability, competition confidence, and technical level ability, with a total of 14 items. Because the present study focused on athletes' perceived performance-related functioning rather than objective competition results, a self-report questionnaire was considered appropriate for assessing these multidimensional aspects of athletic performance. Cronbach's α = 0.887.

The measurement properties of the four instruments were further evaluated using confirmatory factor analysis and internal consistency reliability. As shown in Table 2, all four instruments demonstrated acceptable model fit and satisfactory reliability.

**Table 2:** Reliability and model fit of the measurement instruments.

Instrument	Items (n)	*χ^2^*	*df*	*χ^2^/df*	GFI	AGFI	NFI	RMSEA	Cronbach's α
SM	18	197.553	129	1.531	0.972	0.963	0.970	0.026	0.895
PR	27	490.543	321	1.528	0.955	0.947	0.958	0.026	0.931
CS	18	167.409	132	1.268	0.976	0.969	0.975	0.019	0.897
AP	14	102.157	74	1.381	0.981	0.974	0.981	0.022	0.887

The criteria for model fit were χ^2^*/df* < 3, RMSEA < 0.06, and GFI, AGFI and NFI approaching or exceeding 0.90. Cronbach's α values above 0.70 indicated acceptable reliability. SM, Self-Management; PR, Psychological Resilience; CS, Competitive Spirit; AP, Athletic Performance

### 2.3 Data analysis

Multiple statistical methods were adopted to address the research objectives and test the hypothesized relationships among self-management, psychological resilience, competitive spirit, and athletic performance. SPSS 31.0 was used for data screening, descriptive statistics, and correlation analysis, whereas AMOS 31.0 was used for confirmatory factor analysis (CFA) and structural equation modeling (SEM). Statistical significance was set at *p* < 0.05.

First, to examine common method bias (CMB), a single-factor CFA was conducted using AMOS 31.0. All measurement items were loaded onto one latent factor to construct a single-factor model, thereby testing whether obvious common method bias existed in the measurement data.

Second, regarding the measurement model test, AMOS 31.0 was used to conduct confirmatory factor analysis on the latent variables of self-management, psychological resilience, competitive spirit, and athletic performance, in order to examine the structural validity of the scales. At the same time, Cronbach's α coefficient, composite reliability (CR), and average variance extracted (AVE) were used to examine the internal consistency reliability and convergent validity of each scale.

Finally, to directly address the research hypotheses, SEM was used to estimate the structural paths among self-management, psychological resilience, competitive spirit, and athletic performance. AmosEstimandVB programming combined with the Bootstrap method with 5,000 repeated samples was then used to estimate bias-corrected confidence intervals for the direct, single mediating, and chain mediating effects. If the confidence interval did not include zero, the effect was considered statistically significant.

## 3 Results

### 3.1 Common method bias and model fit tests

To reduce systematic errors that may arise from the same measurement source, this study adopted procedural control measures in the questionnaire design, including anonymous responses, random presentation of items, and interspersed arrangement of different constructs. At the statistical level, single-factor confirmatory factor analysis and comparison with the complete model were conducted for testing (Podsakoff et al., 2003).

The fit indices of the single-factor model were as follows: χ^2^ = 16,969.889, *df* = 2,845, χ^2^*/df* = 5.965, GFI = 0.491, AGFI = 0.463, CFI = 0.542, NFI = 0.497, TLI = 0.529, and RMSEA = 0.080. Compared with the complete multifactor model, χ^2^ = 3,434.622, *df* = 2,830, χ^2^*/df* = 1.214, GFI = 0.899, AGFI = 0.893, CFI = 0.980, NFI = 0.898, TLI = 0.980, and RMSEA = 0.017, the single-factor model showed substantially poorer fit. This indicates that serious common method bias is unlikely to pose a substantial threat to the validity of the research findings (Podsakoff et al., 2003). However, because all variables were collected at the same time point through self-report questionnaires, the possibility of residual common method bias still cannot be completely excluded. Therefore, appropriate caution should be exercised when interpreting the findings, and future research may combine self-report data with objective behavioral indicators or multisource data.

The overall measurement model demonstrated an acceptable fit to the data: χ^2^ = 3,434.622, df = 2,830, χ^2^*/df* = 1.214, GFI = 0.899, AGFI = 0.893, CFI = 0.980, NFI = 0.898, TLI = 0.980, and RMSEA = 0.017. These results supported the adequacy of the overall measurement model for subsequent structural model analysis (Hu and Bentler, 1999; Kline, 2023).

### 3.2 Confirmatory factor analysis

AMOS 31.0 was used to conduct confirmatory factor analysis (CFA) on the four core latent variables, and squared multiple correlations (SMC), composite reliability (CR), and average variance extracted (AVE) were calculated to examine the convergent validity of each latent variable.

The results in Table 3 show that all measurement items loaded significantly on their corresponding factors (*p* < 0.001), with standardized factor loadings exceeding 0.70. The CR values ranged from 0.853 to 0.921, and the AVE values ranged from 0.529 to 0.625, indicating satisfactory convergent validity. More specifically, for self-management, standardized factor loadings ranged from 0.712 to 0.812, with CR values ranging from 0.853 to 0.865 and AVE values ranging from 0.550 to 0.611 across its four dimensions. For athletic performance, standardized factor loadings ranged from 0.737 to 0.817, with CR values ranging from 0.869 to 0.878 and AVE values ranging from 0.573 to 0.625 across its three dimensions. These results confirmed adequate measurement quality of the focal predictor and outcome constructs before hypothesis testing. Psychological resilience and competitive spirit also met the recommended criteria for convergent validity (Fornell and Larcker, 1981).

**Table 3:** Confirmatory factor analysis results.

Latent Variable	Factor	Item	Unstd.	S.E.	*Z*	*p*	Std.	SMC	CR	AVE
SM	SM1	SM1-1	1.000				0.712	0.507	0.859	0.550
		SM1-2	1.060	0.056	18.972	^***^	0.751	0.564		
		SM1-3	1.096	0.055	19.762	^***^	0.786	0.618		
		SM1-4	1.035	0.056	18.325	^***^	0.723	0.523		
		SM1-5	1.029	0.055	18.580	^***^	0.734	0.539		
	SM2	SM2-1	1.000				0.745	0.555	0.865	0.561
		SM2-2	1.054	0.053	19.856	^***^	0.749	0.561		
		SM2-3	1.033	0.053	19.579	^***^	0.739	0.546		
		SM2-4	1.056	0.053	19.941	^***^	0.752	0.566		
		SM2-5	1.029	0.051	20.166	^***^	0.761	0.579		
	SM3	SM3-1	1.000				0.812	0.659	0.853	0.592
		SM3-2	0.953	0.046	20.883	^***^	0.738	0.545		
		SM3-3	0.936	0.043	21.643	^***^	0.762	0.581		
		SM3-4	0.932	0.043	21.717	^***^	0.764	0.584		
	SM4	SM4-1	1.000				0.788	0.621	0.862	0.611
		SM4-2	0.999	0.046	21.791	^***^	0.777	0.604		
		SM4-3	1.026	0.046	22.386	^***^	0.797	0.635		
		SM4-4	0.933	0.044	21.360	^***^	0.763	0.582		
PR	PR1	PR1-1	1.000				0.743	0.552	0.916	0.548
		PR1-2	0.975	0.048	20.165	^***^	0.727	0.529		
		PR1-3	0.959	0.047	20.561	^***^	0.740	0.548		
		PR1-4	0.993	0.047	21.150	^***^	0.760	0.578		
		PR1-5	0.970	0.048	20.306	^***^	0.732	0.536		
		PR1-6	0.970	0.048	20.179	^***^	0.727	0.529		
		PR1-7	0.979	0.048	20.467	^***^	0.737	0.543		
		PR1-8	0.995	0.048	20.927	^***^	0.752	0.566		
		PR1-9	1.040	0.050	20.741	^***^	0.746	0.557		
	PR2	PR2-1	1.000				0.719	0.517	0.913	0.539
		PR2-2	0.992	0.050	19.931	^***^	0.744	0.554		
		PR2-3	1.017	0.050	20.372	^***^	0.761	0.579		
		PR2-4	1.009	0.051	19.861	^***^	0.742	0.551		
		PR2-5	1.029	0.053	19.438	^***^	0.726	0.527		
		PR2-6	1.016	0.054	18.913	^***^	0.707	0.500		
		PR2-7	0.989	0.052	19.174	^***^	0.716	0.513		
		PR2-8	1.050	0.051	20.504	^***^	0.765	0.585		
		PR2-9	1.021	0.053	19.406	^***^	0.725	0.526		
	PR3	PR3-1	1.000				0.744	0.554	0.921	0.565
		PR3-2	0.988	0.047	21.187	^***^	0.757	0.573		
		PR3-3	0.953	0.047	20.388	^***^	0.730	0.533		
		PR3-4	0.963	0.046	20.933	^***^	0.748	0.560		
		PR3-5	1.009	0.047	21.678	^***^	0.773	0.598		
		PR3-6	0.985	0.047	20.761	^***^	0.743	0.552		
		PR3-7	0.975	0.047	20.909	^***^	0.748	0.560		
		PR3-8	1.006	0.046	21.697	^***^	0.773	0.598		
		PR3-9	0.950	0.045	20.901	^***^	0.747	0.558		
CS	CS1	CS1-1	1.000				0.735	0.540	0.881	0.553
		CS1-2	1.033	0.051	20.390	^***^	0.766	0.587		
		CS1-3	0.946	0.050	18.845	^***^	0.709	0.503		
		CS1-4	0.993	0.050	19.904	^***^	0.748	0.560		
		CS1-5	0.991	0.050	19.706	^***^	0.740	0.548		
		CS1-6	1.002	0.049	20.325	^***^	0.763	0.582		
	CS2	CS2-1	1.000				0.757	0.573	0.898	0.558
		CS2-2	0.926	0.046	19.965	^***^	0.728	0.530		
		CS2-3	0.959	0.046	20.710	^***^	0.753	0.567		
		CS2-4	0.972	0.047	20.484	^***^	0.745	0.555		
		CS2-5	0.947	0.046	20.649	^***^	0.751	0.564		
		CS2-6	0.954	0.046	20.620	^***^	0.750	0.563		
	CS3	CS3-1	1.000				0.744	0.554	0.876	0.540
		CS3-2	1.003	0.052	19.385	^***^	0.725	0.526		
		CS3-3	1.007	0.051	19.691	^***^	0.736	0.542		
		CS3-4	1.013	0.052	19.564	^***^	0.732	0.536		
		CS3-5	0.955	0.050	19.253	^***^	0.720	0.518		
		CS3-6	1.010	0.050	20.103	^***^	0.751	0.564		
AP	AP1	AP1-1	1.000				0.737	0.543	0.870	0.573
		AP1-2	1.004	0.050	20.182	^***^	0.766	0.587		
		AP1-3	0.963	0.048	19.992	^***^	0.759	0.576		
		AP1-4	1.024	0.051	19.971	^***^	0.758	0.575		
		AP1-5	1.038	0.052	20.156	^***^	0.765	0.585		
	AP2	AP2-1	1.000				0.742	0.551	0.878	0.591
		AP2-2	0.997	0.048	20.948	^***^	0.780	0.608		
		AP2-3	0.988	0.050	19.812	^***^	0.738	0.545		
		AP2-4	1.021	0.047	21.597	^***^	0.804	0.646		
		AP2-5	1.038	0.050	20.877	^***^	0.777	0.604		
	AP3	AP3-1	1.000				0.759	0.576	0.869	0.625
		AP3-2	1.019	0.046	22.240	^***^	0.817	0.667		
		AP3-3	0.961	0.045	21.361	^***^	0.784	0.615		
		AP3-4	0.970	0.044	21.845	^***^	0.801	0.642		

### 3.3 Correlation and descriptive statistical analysis

Prior to hypothesis testing, preliminary assumption checks were conducted for normality and multicollinearity. The skewness values ranged from −0.573 to −0.381, while the kurtosis values ranged from 0.396 to 1.029, indicating no substantial deviation from normality. The correlations among the study variables ranged from 0.426 to 0.523, with no excessively high correlations observed, suggesting no serious multicollinearity problem (Hair et al., 2019).

Descriptive statistics and correlations among the study variables are presented in Table 4. Significant positive correlations were observed among self-management, psychological resilience, competitive spirit, and athletic performance (*p* < 0.01). In addition, the square root of the AVE for each latent variable exceeded its correlations with the other constructs, supporting adequate discriminant validity (Fornell and Larcker, 1981). Overall, the preliminary analyses indicated that the data were suitable for subsequent structural equation modeling and hypothesis testing.

**Table 4:** Descriptive statistics and correlations among latent variables.

Latent Variable	*M*	SD	Skewness	Kurtosis	AVE	SM	PR	CS	AP
SM	3.500	0.775	−0.538	1.029	0.579	***0.761***			
PR	3.413	0.781	−0.562	0.928	0.551	0.473^**^	***0.742***		
CS	3.498	0.776	−0.573	0.979	0.550	0.426^**^	0.483^**^	***0.742***	
AP	3.299	0.820	−0.381	0.396	0.596	0.497^**^	0.523^**^	0.512^**^	***0.772***

^**^p < 0.01; Italicized bold values on the diagonal represent the square roots of AVEs.

### 3.4 Path effect model analysis

A structural equation model was constructed based on AMOS, and the Bootstrap method with 5,000 repeated samples was used to test the direct and mediating effects.

#### 3.4.1 Path coefficients

Figure 2 presents the standardized coefficients of each path in the model. The path from self-management to athletic performance, path **a**, was β = 0.26, *p* < 0.001. The path from self-management to psychological resilience, path **b**, was β = 0.69, *p* < 0.001. The path from psychological resilience to competitive spirit, path **c**, was β = 0.58, *p* < 0.001. The path from competitive spirit to athletic performance, path **d**, was β = 0.39, *p* < 0.001. The path from self-management to competitive spirit, path e, was β = 0.23, *p* < 0.01. The path from psychological resilience to athletic performance, path f, was β = 0.32, *p* < 0.01.

**Figure 2:**
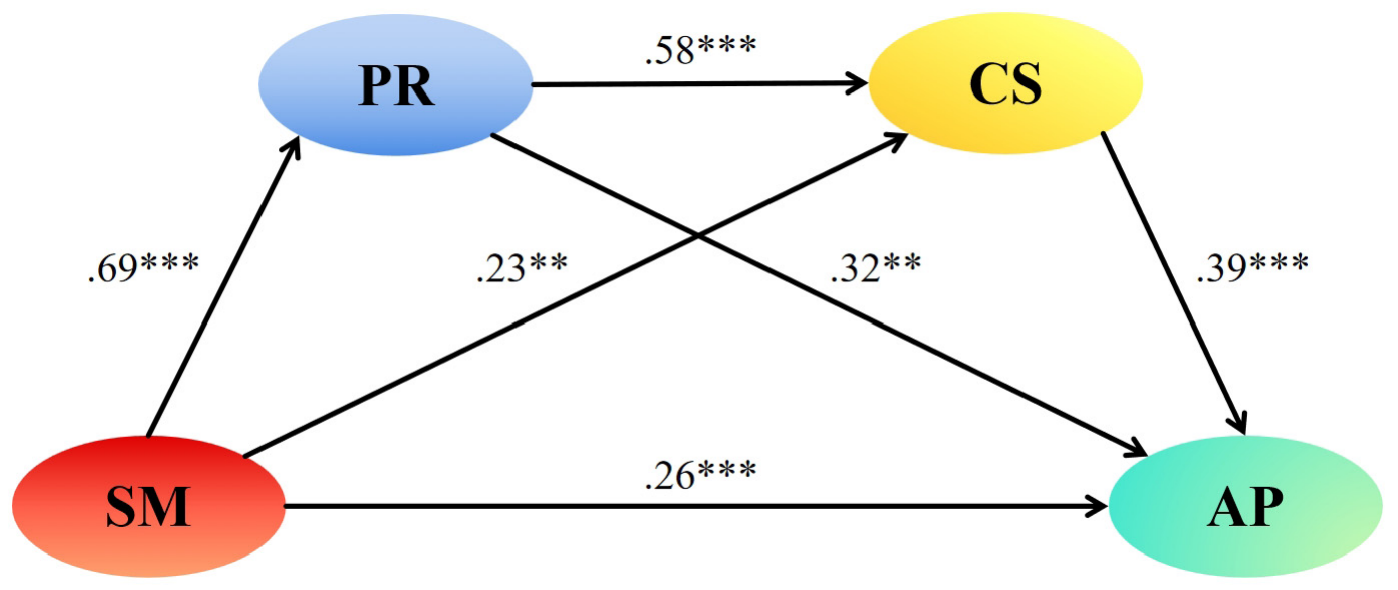
Model and Path Coefficients. **p < 0.01, ***p < 0.001. SM, Self-Management; PR, Psychological Resilience; CS, Competitive Spirit; AP, Athletic Performance effect a: SM → AP; effect b: SM → PR; effect c: PR → CS; effect d: CS → AP; effect e: SM → CS; effect f: PR → AP.

#### 3.4.2 Model effect analysis

In AMOS, the paths of the direct and mediating effects were coded using the letters a–f. An AmosEstimandVB programming document was created, and coding instructions were processed according to the effect categories corresponding to hypotheses H1–H4. Furthermore, the model was estimated and tested, and the final results of the direct and mediating effects are presented in Table 5.

**Table 5:** Effect analysis.

	Hypothesis/Effect	β	S.E.	Lower	Upper	*p*
H1	Direct effect(a)	0.234^*^	0.096	0.035	0.418	0.0312
H2	Indirect effect(bf)	0.203^*^	0.099	0.044	0.421	0.0223
H3	Indirect effect(ed)	0.080^*^	0.054	0.008	0.234	0.0296
H4	Serial mediation effect(bcd)	0.141^***^	0.070	0.059	0.325	0.0007
	Total	0.658^**^	0.066	0.528	0.794	0.0011

Path a = SM → AP; Path b = SM → PR; Path c = PR → CS; Path d = CS → AP; Path e = SM → CS; Path f = PR → AP. The indirect effect through PR was calculated as b × f; the indirect effect through CS was calculated as e × d; the serial mediation effect was calculated as b × c × d. ^*^p < 0.05, ^**^p < 0.01, ^***^p < 0.001. The results showed that the total effect of the model was 0.658, SE = 0.066, 95% CI [0.528, 0.794], excluding zero, indicating that the total effect was significant. For the direct effect path of H1, effect “a” was β = 0.234, SE = 0.096, 95% CI [0.035, 0.418], supporting H1. For the mediating effect path of H2, effect “bf” was β = 0.203, SE = 0.099, 95% CI [0.044, 0.421], supporting H2. For the mediating effect path of H3, effect “ed” was β = 0.080, SE = 0.054, 95% CI [0.008, 0.234], supporting H3. For the chain mediating effect path of H4, effect “bcd” was β = 0.141, SE = 0.070, 95% CI [0.059, 0.325], supporting H4.

## 4 Discussion

Based on data from 772 Chinese track and field athletes, this study examined the pathways through which self-management influences athletic performance. The results showed that self-management had a significant positive effect on athletic performance. Meanwhile, psychological resilience and competitive spirit each played a significant positive mediating role between self-management and athletic performance. In addition, psychological resilience and competitive spirit formed a significant positive chain mediating pathway.

### 4.1 Self-management and athletic performance: the direct role of behavioral regulation

Self-management was positively associated with athletic performance among Chinese track and field athletes, supporting H1. This finding is consistent with recent evidence showing that athletes with stronger self-management tend to report better performance-related outcomes. For example, Nam et al. (2024) found that self-management was significantly associated with perceived performance among judo athletes, while Kim and Kim (2024) reported that physical, training, and mental management were significantly related to performance among university athletes. Similar findings have also been observed in team-sport athletes, where self-management was positively associated with athletic performance (Kim and Kim, 2021).

The present finding extends this evidence to Chinese track and field athletes, a context in which effective self-regulation may be particularly important because athletes must continually manage training load, recovery, psychological state, and performance goals across prolonged training and competition cycles. This interpretation is also consistent with recent research on distance running showing that successful performance involves ongoing self-regulatory processes, including goal monitoring, adjustment, and adaptive goal revision during competition (Jackman et al., 2024). Accordingly, the present result suggests that self-management should be understood not merely as a routine training habit, but as a broader behavioral-regulatory resource associated with athletes' perceived performance.

### 4.2 Mediating role of psychological resilience: from self-management to psychological adaptation

Self-management was positively associated with psychological resilience, which in turn was positively associated with athletic performance, supporting H2. This pattern is consistent with recent sport research suggesting that stronger self-regulatory capacities are closely related to athletes' ability to adapt to stress and adversity. Kim and Choi (2023), for example, reported a positive association between self-management and resilience among paragliding athletes, while Proietti et al. (2024) showed that self-regulation training can strengthen athletes' resilience under stressful conditions. These findings are also consistent with the broader view of sporting resilience as a dynamic adaptive process shaped by athletes' cognitive, emotional, and behavioral regulation (Gupta and McCarthy, 2022).

The association between psychological resilience and athletic performance is likewise supported by recent evidence. Chrétien et al. (2024) found that elite athletes with stronger resilience profiles tended to report more favorable performance-related outcomes, and a recent systematic review and meta-analysis further confirmed a robust positive relationship between resilience and athletic performance across different sport contexts (Mei et al., 2026). Together, these studies suggest that resilience is not merely a protective resource for coping with adversity, but also an important psychological capacity associated with maintaining effective performance under demanding competitive conditions.

The present study extends this literature by integrating these two relationships within a single indirect pathway. Rather than treating resilience only as an independent correlate of athletic performance, the findings suggest that it may serve as an adaptive psychological resource linking athletes' self-management with their perceived performance. For track and field athletes, the continuous regulation of training, recovery, emotional responses, and competitive demands may contribute to stronger adaptive capacity, which may in turn support more stable performance-related functioning.

### 4.3 Mediating role of competitive spirit: the transformation of self-management into competitive motivation

Self-management was positively associated with competitive spirit, which was subsequently associated with better athletic performance, supporting H3. This finding is broadly consistent with contemporary research highlighting the close interplay between self-regulatory functioning and competition-oriented motivation in sport. Bugten et al. (2021) showed that the need to compete is embedded within a broader achievement-oriented motivational system involving self-efficacy, grit, anxiety, and flow, while more recent studies have linked achievement-oriented motivational dispositions with athletes' self-management and sustained engagement in training (Kim, 2022; Lee and Kwon, 2024). Although competitive spirit is conceptually distinct from achievement-goal orientation, these findings collectively suggest that effective behavioral regulation and competitive striving are closely interconnected within the athletic context.

The association between competitive spirit and athletic performance is also consistent with recent evidence showing that achievement- and competition-oriented motivation is relevant to performance-related functioning. Kim (2023) reported that stronger achievement-goal orientation was associated with more favorable performance among adolescent basketball players, while Lochbaum and Sisneros (2024), in a systematic review and meta-analysis, demonstrated that achievement-goal orientations are meaningfully related to sport performance across a range of competitive settings. Taken together, this literature supports the view that athletes who maintain clearer competitive goals and a stronger orientation toward achievement may be better able to sustain effort, commitment, and performance-directed behavior under demanding training and competition conditions.

The present study extends this line of research by positioning competitive spirit not merely as an independent motivational characteristic, but as a potential motivational pathway through which self-management is associated with athletic performance. For track and field athletes, the systematic regulation of training, recovery, physical condition, and psychological state may help preserve goal clarity and competitive commitment across prolonged and repetitive training cycles. In this sense, self-management may do more than organize athletes' behavior; it may also provide the regulatory foundation through which competitive motivation is maintained and translated into performance-related functioning. This interpretation broadens the role of self-management from a behavioral control process to a mechanism potentially linked with the development and maintenance of athletes' competitive striving.

### 4.4 Chain mediating role of psychological resilience and competitive spirit: the transformation from psychological resources to competitive motivation

Psychological resilience and competitive spirit sequentially mediated the association between self-management and athletic performance, supporting H4. This finding suggests that self-management may be linked to performance through a progressive process involving behavioral regulation, psychological adaptation, and competitive motivation. Effective management of training, recovery, physical condition, and psychological states may enhance athletes' sense of control and strengthen their capacity to cope with pressure, setbacks, and performance fluctuations. In turn, stronger psychological resilience may help athletes maintain goal focus, confidence, and competitive engagement under demanding conditions, thereby supporting stronger competitive striving. This interpretation is consistent with recent evidence linking self-management with resilience and with contemporary perspectives that conceptualize sporting resilience as a dynamic adaptive process involving cognitive, emotional, and behavioral regulation rather than a fixed personal trait (Gupta and McCarthy, 2022; Kim and Choi, 2023).

More importantly, the present study extends previous research by integrating psychological resilience and competitive spirit within a single sequential mechanism. Whereas resilience and competition-oriented motivation have often been examined separately in relation to athletic performance, the present findings suggest that adaptive psychological resources may provide an important foundation for sustaining competitive motivation in challenging sport environments. Recent research has similarly emphasized the relevance of resilience and achievement-oriented motivation to athletes' performance-related functioning (Chrétien et al., 2024; Lochbaum and Sisneros, 2024). Although competitive spirit is conceptually distinct from achievement-goal orientation, these findings collectively support the idea that athletes' behavioral self-regulation may be associated with performance through a sequence of strengthened adaptation and competitive striving. Thus, the contribution of the present study lies in showing how behavioral regulation, psychological adaptation, and competitive motivation may operate as an interconnected pathway associated with athletic performance.

## 5 Limitations and future research

Although this study constructed and tested a chain mediation model of the influence of self-management on track and field athletes' athletic performance, and revealed the mechanisms of psychological resilience and competitive spirit within this relationship, several limitations remain.

First, this study adopted a cross-sectional design, and all variables were measured at the same time point. Therefore, caution is needed when interpreting the temporal order and causal relationships among the variables. Although this study constructed the mediating pathways based on theoretical assumptions, the results are more appropriately understood as indirect associations in a statistical sense. Future research may adopt longitudinal tracking, cross-lagged models, or experimental intervention designs to further verify the findings of this study.

Second, data were collected using self-report questionnaires. Although common method bias was controlled and tested, the results may still be affected by subjective judgment, recall bias, and social desirability effects. Future research may combine multiple sources of data, such as competition results, coach evaluations, and training monitoring, to further improve the objectivity and ecological validity of measurement.

Third, the sample was substantially imbalanced by gender and event type, with a predominance of male athletes and sprinters. Given possible gender- and event-related differences in competitive orientation and performance-related psychological characteristics, this distribution may limit the generalizability of the findings to female athletes and to athletes in other track and field disciplines. In addition, 33.4% of the participants were non-ranked athletes, defined here as athletes without an official athletic-level classification. Because detailed competitive history was not separately assessed and athletic performance was measured using self-report rather than objective indicators such as competition times, personal bests, or official rankings, the findings for the non-ranked subgroup should be interpreted with caution. Future studies should recruit more balanced samples across gender and event categories and incorporate objective performance indicators and more detailed competition-history information.

## 6 Conclusion

Taking Chinese track and field athletes as the research participants, this study constructed and tested a mediation model of the influence of self-management on athletic performance based on self-regulation theory and relevant perspectives in sport psychology. The results showed that self-management had a significant positive effect on athletic performance; the higher the athletes' level of self-management, the better their athletic performance. Psychological resilience and competitive spirit each played a significant positive mediating role between self-management and athletic performance, and together they formed a significant positive chain mediating pathway. Self-management can not only directly promote athletic performance, but also improve athletic performance by enhancing psychological resilience and strengthening competitive spirit.

This study further indicates that the formation of track and field athletes' athletic performance does not depend only on physical fitness, technical skills, and sport-specific training, but is also jointly influenced by self-regulation ability, psychological adaptation ability, and competitive goal orientation. For track and field athletes, long-term, repetitive, and high-intensity training can easily lead to physical fatigue, psychological pressure, and performance fluctuations. If athletes lack good self-management ability, it is difficult for them to continuously maintain a stable training state and competition performance. Therefore, self-management should be regarded as an important psychological and behavioral foundation for improving athletic performance, rather than merely a part of athletes' daily life management or training discipline. At the same time, the mediating roles of psychological resilience and competitive spirit between self-management and athletic performance also indicate that the effect of self-management on athletic performance does not follow a single pathway, but is further realized through the sequential effects of psychological adaptation ability and competitive motivation.

At the practical level, the findings of this study suggest that coaches and sport team managers should incorporate the cultivation of self-management ability, psychological resilience, and competitive spirit into the daily training system of track and field athletes. Specifically, training plan management, phased goal setting, training log recording, recovery status monitoring, and post-competition feedback can be used to help athletes develop stable training habits and self-regulation ability. Meanwhile, pressure-situation simulation, review of failure experiences, and emotional regulation training can be used to improve athletes' psychological recovery ability when facing setbacks, injuries, competition losses, and performance fluctuations. In addition, when cultivating competitive spirit, coaches should avoid simply emphasizing competition results. Instead, they should guide athletes to establish positive, healthy, and stable competitive goals, enabling them to further transform self-management and psychological resilience into sustained training commitment, competition confidence, and competitive motivation.

## Data Availability

The datasets generated and/or analyzed during the current study are available from the corresponding author upon reasonable request.

## References

[B1] Bugten J. B. Lugo R. G. Steptoe K. (2021). Validation of the need for competing inventory. Front. Psychol. 12:721903. doi: 10.3389/fpsyg.2021.721903 PMC8517083 34659033

[B2] Choi J. B. (2014). The Relationship Between Athletic Performance and Psychosocial Factors in Taekwondo Kyorugi Athletes. (Doctoral dissertation). Woosuk University, Wanju-gun.

[B3] Chrétien A. Hayotte M. Vuillemin A. Longueville F. D. A. (2024). Resilience profiles of elite athletes and their associations with health-related behaviors, wellbeing, and performance: a latent profile analysis. Psychol. Sport Exerc. 74:102689. doi: 10.1016/j.psychsport.2024.102689 38901549

[B4] Fletcher D. Sarkar M. (2012). A grounded theory of psychological resilience in Olympic champions. Psychol. Sport Exerc. 13, 669–678. doi: 10.1016/j.psychsport.2012.04.007

[B5] Fornell C. Larcker D. F. (1981). Evaluating structural equation models with unobservable variables and measurement error. J. Mark. Res. 18, 39–50. doi: 10.1177/002224378101800104

[B6] Gill D. L. Deeter T. E. (1988). Development of the sport orientation questionnaire. Res. Q. Exerc. Sport 59, 191–202. doi: 10.1080/02701367.1988.10605504

[B7] Golzar J. Noor S. Tajik O. (2022). Convenience sampling. Int. J. Educ. Lang. Stud. 1, 72–77.

[B8] Gupta S. McCarthy P. J. (2022). The sporting resilience model: a systematic review of resilience in sport performers. Front. Psychol. 13:1003053. doi: 10.3389/fpsyg.2022.1003053 PMC9811683 36619099

[B9] Hair J. F. Black W. C. Babin B. J. Anderson R. E. Black W. C. Anderson R. E. (2019). Multivariate Data Analysis, 8^*th*^*Edn*. Hampshire: Cengage Learning EMEA.

[B10] Hosseini S. A. Besharat M. A. (2010). Relation of resilience whit sport achievement and mental health in a sample of athletes. Procedia Soc. Behav. Sci. 5, 633–638. doi: 10.1016/j.sbspro.2010.07.156

[B11] Hu L. T. Bentler P. M. (1999). Cutoff criteria for fit indexes in covariance structure analysis: conventional criteria vs new alternatives. Struct. Equ. Model. A Multidiscip. J. 6, 1–55. doi: 10.1080/10705519909540118

[B12] Huh J. H. (2003). Development and validation of athletes' self-management questionnaire ASMQ. Korean J. Sport Psychol. 14, 95–109.

[B13] Jackman P. C. Whitehead A. E. Swann C. Brick N. E. (2024). Self-regulatory processes in goal striving during excellent distance-running performances: a qualitative study. Psychol. Sport Exerc. 70:102516. doi: 10.1016/j.psychsport.2023.102516 38065659

[B14] Kim B.-J. (2022). The relationship between achievement-goal orientation, self-management, and training commitment among middle school athletes. J. Sport All Rec. 88, 239–251. doi: 10.51979/KSSLS.2022.04.88.239

[B15] Kim B.-J. (2023). Relationship between achievement goal orientation, athletic passion, training flow and performance of middle and high school basketball players. Korean J. Sport Sci. 21, 563–575. doi: 10.46669/kss.2023.21.1.049

[B16] Kim B. J. (2003). Measuring self-management practices in Korean athletes. Korean J. Sport Sci. 14, 125–140.

[B17] Kim H.-H. Choi J.-I. (2023). The effect of self-management of paragliding athletes on resilience. Korean J. Sport Sci. 21, 1121–1133. doi: 10.46669/kss.2023.21.4.095

[B18] Kim H. D. Cruz A. B. (2021). Psychological influence of self-management on exercise self-confidence, satisfaction, and commitment of martial arts practitioners in Korea: a meta-analytic approach. Front. Psychol. 12:691974. doi: 10.3389/fpsyg.2021.691974 PMC8200399 34135839

[B19] Kim J. W. Kim B. J. (2021). Verifying the mediated effect of athletic performance in the relationship between the self-management and the exercise commitment of team sports athletes. J. Sport All Rec. 86, 321–331. doi: 10.51979/KSSLS.2021.10.86.321

[B20] Kim Y.-S. Kim T.-H. (2024). Effects of athletes' self-management and competitive state anxiety on performance. J. Korea Entertain. Ind. Assoc. 18, 97–106. doi: 10.21184/jkeia.2024.11.18.8.97

[B21] Kline R. B. (2023). Principles and Practice of Structural Equation Modeling. New York, NY: Guilford publications.

[B22] Lee H. Kwon S. (2024). The relationship between julsil and self-management in male adolescent athletes: the moderating effects of achievement goal orientation. Korean J. Sport Sci. 35, 588–599. doi: 10.24985/kjss.2024.35.4.588

[B23] Lochbaum M. Sisneros C. (2024). Situational and dispositional achievement goals and measures of sport performance: a systematic review with a meta-analysis. Sports 12:299. doi: 10.3390/sports12110299 PMC11598045 39590901

[B24] Mei Z. Cai C. Lam C. Wang T. Jiang H. Luo S. (2026). The association between resilience and athletic performance: findings from a systematic review and meta-analysis of observational studies. BMC Psychol 14:526. doi: 10.1186/s40359-026-04247-5 PMC13077836 41792850

[B25] Nam K. Kim C. Yoon S. (2024). Relationship between judo coaches' authentic leadership and judo athletes' perceived performance: mediating effect of self-management. Int. J. Sports Sci. Coach. 19, 19–32. doi: 10.1177/17479541231190036

[B26] Podsakoff P. M. MacKenzie S. B. Lee J. Y. Podsakoff N. P. (2003). Common method biases in behavioral research: a critical review of the literature and recommended remedies. J. Appl. Psychol. 88, 879–903. doi: 10.1037/0021-9010.88.5.879 14516251

[B27] Proietti G. Borozan M. Chaigneau A. Cannito L. Palumbo R. Thouvarecq R. . (2024). Self-regulation training improves stress resilience in elite pre-pubescent female gymnasts. Front. Psychol. 15:1341437. doi: 10.3389/fpsyg.2024.1341437 PMC11076701 38721324

[B28] Reinebo G. Alfonsson S. Jansson-Fröjmark M. Rozental A. Lundgren T. (2024). Effects of psychological interventions to enhance athletic performance: a systematic review and meta-analysis: G. Reinebo et al. Sports Med. 54, 347–373. doi: 10.1007/s40279-023-01931-z PMC10933186 37812334

[B29] Sarkar M. Fletcher D. (2014). Psychological resilience in sport performers: a review of stressors and protective factors. J. Sports Sci. 32, 1419–1434. doi: 10.1080/02640414.2014.901551 24716648

[B30] Shin J. T. Kim S. H. Park Y. S. (2009). Development of a competitive tenacity questionnaire for top-level Korean athletes. J. Sport Leis. Stud. 36, 755–772. doi: 10.51979/KSSLS.2009.05.36.755

[B31] Shin W. Y. Kim M. K. Kim J. H. (2009). Development and validation of the resilience test index. Korean J. Youth Stud. 20, 105–131.

[B32] Toering T. T. Elferink-Gemser M. T. Jordet G. Visscher C. (2009). Self-regulation and performance level of elite and non-elite youth soccer players. J. Sports Sci. 27, 1509–1517. doi: 10.1080/02640410903369919 19967593

[B33] Young B. W. Wilson S. G. Hoar S. Bain L. Siekańska M. Baker J. (2023). On the self-regulation of sport practice: moving the narrative from theory and assessment toward practice. Front. Psychol. 14:1089110. doi: 10.3389/fpsyg.2023.1089110 PMC10086193 37057149

[B34] Zimmerman B. J. (2000). “Attaining self-regulation: a social cognitive perspective,” in Handbook of Self-regulation. Cambridge, MA: Academic Press, 13–39. doi: 10.1016/B978-012109890-2/50031-7

